# A meta-analysis of the literature evaluating the impact of vitamin D on female fertility and ovarian reserve function

**DOI:** 10.1590/1980-220X-REEUSP-2025-0382en

**Published:** 2026-02-02

**Authors:** Shanfei Zhao, Wenling Zheng, Guanyun Long, Guanglin Liang

**Affiliations:** 1 Maoming People ’s Hospital, Center of Reproductive Medicine, Guangdong Province, China.

**Keywords:** Vitamin D, Infertility, Polycystic Ovarian Syndrome, Ovulation; Pregnancy, Anti-Mullerian Hormone, Vitamina D, Infertilidade, Síndrome do Ovário Policístico, Ovulação, Gravidez, Hormônio Antimülleriano

## Abstract

**Objective:**

The current analysis study aims to estimate the role of vitamin D levels and supplementation on fertility reservation expressed as ovulation rate, pregnancy rate, and anti-Müllerian hormone (AMH).

**Method:**

A systematic literature review was conducted, and 2111 females were recruited to the current study. Inclusion criteria of the current study included comparative studies evaluating the role of vitamin D status and vitamin D supplementation on fertility parameters. Included studies published till December 2023. Continuous mode with random effect, were used for the analysis of data.

**Results:**

Twenty-two studies, conducted on the impact of vitamin D on fertility parameters for females, were recruited in the current study. Vitamin D supplementation showed a significant enhancement in several parameters, including vitamin D level, number of dominant follicles, Ovulation rate, and pregnancy rate (MD = –2.3295% CI [–3.07, –1.56], MD = 0.81 95% CI [0.27, 1.35], MD = 0.81 95% CI [0.17, 1.45], and MD = 0.64 95% CI [0.20, 1.07], respectively). On the other hand, there was no significant impact on AMH levels.

**Conclusion:**

Findings of current studies indicate a beneficial impact of vitamin D therapy on fertility outcomes regarding the number of dominant follicles, ovulation rate, and pregnancy rate.

## RESUMO

**Objetivo:**

O presente estudo de meta-análise teve como objetivo estimar o papel dos níveis de vitamina D e da suplementação de vitamina D na reserva reprodutiva, expressa pela taxa de ovulação, taxa de gravidez e pelo hormônio anti-Mülleriano (AMH).

**Método:**

Foi realizada uma revisão sistemática da literatura, incluindo um total de 2.111 mulheres no presente estudo. Os critérios de inclusão abrangeram estudos comparativos que avaliaram o estado de vitamina D e a suplementação de vitamina D em parâmetros de fertilidade. Foram incluídos estudos publicados até dezembro de 2023. Para a análise dos dados utilizou-se um modelo em modalidade contínua com efeitos aleatórios.

**Resultados:**

Foram recrutados 22 estudos que investigaram o impacto da vitamina D nos parâmetros de fertilidade feminina. A suplementação de vitamina D mostrou um aumento significativo em vários parâmetros, incluindo níveis de vitamina D, número de folículos dominantes, taxa de ovulação e taxa de gravidez (MD = –2.3295; 95% CI [–3.07, –1.56]; MD = 0.81; 95% CI [0.27, 1.35]; MD = 0.81; 95% CI [0.17, 1.45]; e MD = 0.64; 95% CI [0.20, 1.07], respetivamente). Por outro lado, não se observou efeito significativo sobre os níveis de AMH.

**Conclusão:**

Os achados do presente estudo indicam um impacto benéfico da terapia com vitamina D nos desfechos de fertilidade, nomeadamente no número de folículos dominantes, na taxa de ovulação e na taxa de gravidez.

## INTRODUCTION

Infertility affects 75% of women with polycystic ovarian syndrome (PCOS), making it the most common endocrine disorder affecting reproductive-aged women^(1)^. Monitoring ovulation, stimulating ovulation, performing ovarian perforation, and utilizing assisted reproductive technology are the main current ways to manage infertility in PCOS^(2)^. Several nutrients, such as minerals, vitamins, and unsaturated fatty acids have also attracted a lot of attention when used as an adjuvant^(3)^. Vitamin D deficiency is a serious issue; it affects 67–85% of PCOS women^(4)^. Insulin resistance and metabolic syndrome can develop in PCOS patients. Vitamin D insufficiency may contribute to this^(5)^. Pregnant women with PCOS are having a slight upward trend in research on the efficacy of vitamin D supplements. Vitamin D ’s protective effects against oxidative stress and its role in regulating calcium ion levels—which are critical for preserving the resting potential of cells—are the reasons behind this trend. The processes of fertilization, cleavage, implantation, and placental development can all benefit from these effects^(6)^.

New studies in both animals and humans suggests that vitamin D is involved in the physiology of female reproduction^(7)^, although the specific mechanism is not well understood. Rats with insufficient dietary vitamin D experience a 75% decline in fertility, accompanied by a 30% decrease in the number of offspring and hindered growth of newborns^(8)^. Vitamin D receptor knockout mice exhibited reduced bone formation and stunted growth, as well as an underdeveloped uterus and decreased follicle development^(9,10)^. The mice had hypergonadotropic hypogonadism, which was supplemented by reduced CYP19 gene expression and aromatase activity. This suggests that vitamin D might have a starring role in estrogen production and the expression of the aromatase gene^(9,10)^. Mice with a receiving 25-hydro-xyvitamin D 1a-hydroxylase exhibit comparable abnormalities, such as an underdeveloped uterus, decreased growth of ovarian follicles, and faulty formation of corpus luteum. Remarkably, administering a diet rich in calcium and/or phosphorus effectively restores the fertility of these animals. This indicates that infertility in these models is a result of low levels of calcium and/or phosphorus caused by a deficiency in vitamin D^(11,12)^. When vitamin D was applied to ovarian cells in a laboratory setting, it resulted in an enhanced production of the sex hormones progesterone, estrogen, and estrone^(13)^. Furthermore, the human placenta had an increase in estrogen and progesterone synthesis when exposed to 1,25-dihydroxyvitamin D3^(14)^. Furthermore, an large number of studies indicates that there is a correlation between vitamin D insufficiency and other symptoms of PCOS, such as anovulation, hyperandrogenism, and insulin resistance^(15)^. It is worth noting that women with PCOS are more likely to have a deficiency in vitamin D^(16,17)^. Studies have demonstrated that supplementing with vitamin D can enhance menstrual cyclicity, reduce hyperandrogenism, and improve metabolic factors associated with this syndrome^(18,19)^. This suggests that vitamin D directly benefits female fertility.

Nevertheless, the existing findings on the impact of vitamin D supplementation on pregnancy rates lack consistency. Several studies have demonstrated different findings regarding the impact of vitamin D supplementation on pregnancy rates in females with PCOS. Some studies have indicated a positive effect, while others have found no substantial benefit^(20)^. This study aims to systematically gather and analyze existing evidence on the use of vitamin D as a supplementary treatment for infertility in females. The objective of the current study analysis is to determine whether vitamin D can improve pregnancy outcomes such as pregnancy rate, ovulation rate, anti-Müllerian hormone (AMH) level, and Number of dominant follicles in females and establish a foundation for clinical medication.

## METHOD

### Study Design

**Figure 1:**
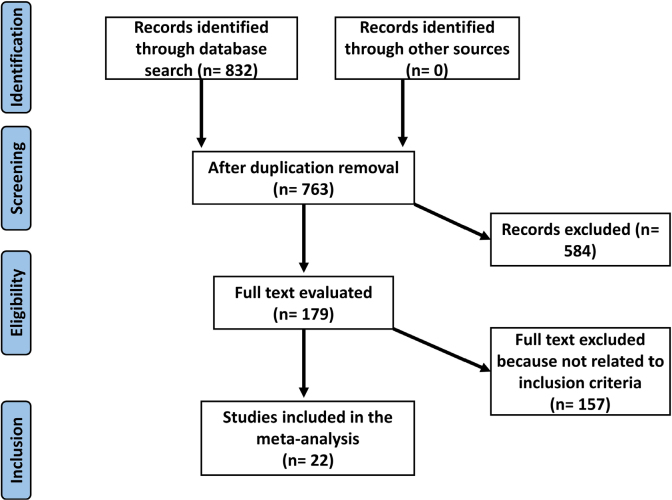
Diagram representing the study procedure – Guangdong, China, 2025.

The epidemiological declaration was the utilized in the present meta-analysis, which encompassed studies that tracked a prearranged study technique^(21)^. Several scientific databases were used to obtain and analyze data from the recruited studies. These scientific databases included OVID, Cochrane Library, PubMed, Embase database, and Google Scholar. The inclusion criteria were followed precisely^(22)^. The entire study sequence is shown in Figure 1.

### Eligibility and Inclusion

Included studies should be oriented to evaluate the role of vitamin D levels and supplementation on fertility reservation expressed as ovulation rate, pregnancy rate, and anti-Müllerian hormone. Only articles that specifically evaluated the interventions affected ovulation rate, pregnancy rate, vitamin D levels, and how vitamin D levels and supplementation affected fertility reserve, as measured by dominant follicle count, ovulation rate, pregnancy rate, and anti-Müllerian hormone level, were included in the sensitivity analysis^(23)^.

### Inclusion Criteria:

1. The acceptable study design to be involved in the current study should be a comparative study published by December 2024.
2. Studies of females who received vitamin D supplemental therapy compared to control females have pre- and post-serum levels of AMH and vitamin D.

### The Exclusion Criteria Were:

1. Articles that were unable to convey the results of comparison between different interventions in a suitable manner, such as interquartile range or median. Studies should always provide their data as a mean (SD) or event/total.
2. In case the study was in the form of letters, books, review articles, or book chapters.

### Identification

First, up until December 2024, we used a combination of several keywords and comparable words for Vitamin D supplementation, vitamin D level, AMH, follicle-stimulating hormone (FSH), fertility, ovulation rate, pregnancy rate, PCOS, and follicles^(24)^. A protocol of search strategies was defined in accordance with the PICOS principle as follows: P (population), females with fertility-related issues, I (intervention/exposure); vitamin D supplementation, C (comparison); intervention group with control group or before and after intervention comparison, O (outcome): vitamin D level, AMH level, ovulation rate, pregnancy rate, number of dominant follicles; S (study design): interventional comparative studies.

Using these and associated keywords, the authors searched Google Scholar, PubMed, Cochrane Library, Embase, and OVID up until December 2024. Following a review of the titles and abstracts included in the reference management program, any paper failing to address and assess the impact of vitamin D on reproductive parameters was excluded^(25)^.

### Screening

A number of criteria were used to filter the data. These included the following: the first author ’s surname, the year of publication, the country of study, the type of study, the length of the study, demographic information, clinical and treatment characteristics, the total number of subjects, the methods used, the information source, and the outcomes. Two separate authors blindly examined the selected papers ’ methodological quality and checked each study for possible bias^(26)^.

Each piece of included research was evaluated for potential bias using a author manager, and the results were ranked from low to high. Each study underwent a methodological examination by two separate authors^(27)^.

### Statistical Analysis

In this meta-analysis, we used a random effect with continuous model to compute the mean difference (MD) with a 95 percent confidence interval (CI). 24 This study used two-tailed testing to get all p-values. Graphs and statistical analyses were created using Jamovi. Using the constrained maximum-likelihood estimator, the level of heterogeneity (tau^2^) was calculated. The I^2^ index was computed, which is a numerical value with a range from 0 to 100 and is conveyed in the form of Forest plots. The heterogeneity level was shown by percentages ranging from 0% to 100% and it was also expressed by percentages indicating no (<25%), low (<50%), moderate (<75%), and high levels of heterogeneity. Begg ’s and Egger tests were used to conduct quantitative analysis on publication bias, and the presence of publication bias was deemed to be present if p > 0.05.

## RESULTS

**Figure 2:**
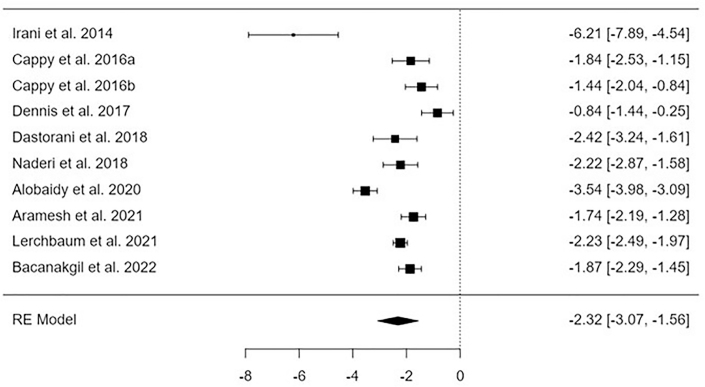
Forest plot indicating the influence of vitamin D therapy on the level of serum vitamin D – Guangdong, China, 2025.

**Figure 3:**
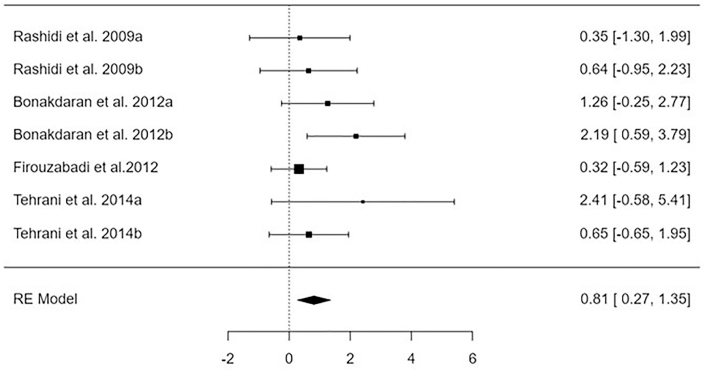
Forest plot indicating the influence of vitamin D therapy on the number of dominant follicles – Guangdong, China, 2025.

**Figure 4:**
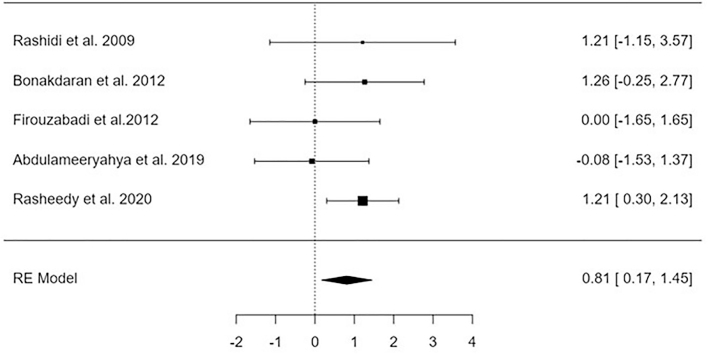
Forest plot indicating the influence of vitamin D therapy on ovulation rate – Guangdong, China, 2025.

**Figure 5:**
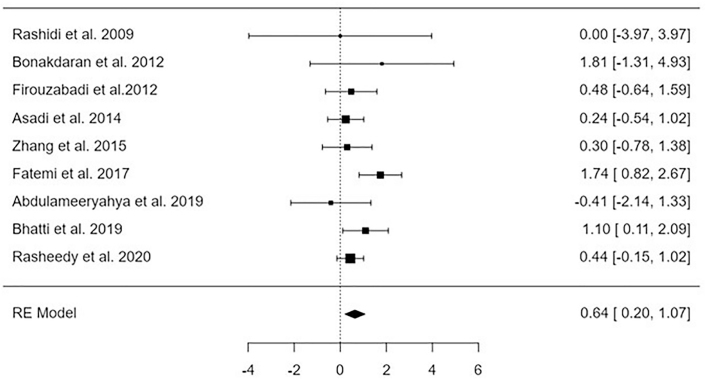
Forest plot indicating the influence of vitamin D therapy on pregnancy rate – Guangdong, China, 2025.

After reviewing 2111 relevant studies, 22 articles from the period of 2009 to 2023 were included in the meta-analysis since they were consistent with the inclusion criteria^(20,28,29,30,31,32,32,33,34,35,36,37,38,39,40,41,42,43,44,45,46,47,48)^. The results of these investigations are compiled in Figures 2-5.

### Vitamin D Supplementation Influenceing Serum Levels of Vitamin D

Analysis of findings extracted from 9 studies evaluating the influence of vitamin D supplement on the level of vitamin D compared to baseline levels before initiation of therapy. Impact of Vitamin D has been revealed to be significant (p < 0.001) on serum levels of vitamin D post-treatment compared with baseline values, MD = –2.32, 95%CI [–3.07, –1.56], I2 = 95.2% as shown in Figure 2. Asymmetry of funnel plot was indicated by the regression test (p = 0.08), while the rank correlation did not indicate publication bias (p = 0.73).

### Number of Dominant Follicles

Analysis of findings extracted from 7 papers assessing the influence of vitamin D supplementation on the number of dominant follicles compared to the control (group of females not receiving the vitamin D supplementation). The impact of Vitamin D has been revealed to be significant (p < 0.01) on the number of dominant follicles, expressed as a high number of dominant follicles for females receiving vitamin D supplementation compared with control, MD = 0.81, 95%CI [0.27, 1.35], I2 = 1.18% as shown in Figure 3. The regression test and the rank correlation indicated no publication bias, p = 0.1, p = 0.24, respectively.

### Ovulation Rate

Analysis of findings extracted from 5 studies assessing the influence of vitamin D supplements on ovulation rate compared to control. The impact of Vitamin D has been revealed to be significant (p = 0.01) on the Ovulation rate, expressed as a high ovulation rate for females receiving vitamin D supplementation compared with control, MD = 0.81, 95%CI [0.17, 1.45], I2 = 5.1% as shown in Figure 4. The regression test and the rank correlation indicated no publication bias, p = 0.52, p = 0.82, respectively.

### Pregnancy Rate

Analysis of findings extracted from 9 studies assessing the impact of vitamin D supplements on pregnancy rate compared to that of controls. Impact of Vitamin D was significant (p < 0.01) on the pregnancy rate, expressed as a high pregnancy rate for females receiving vitamin D supplementation compared with control, MD = 0.46, 95%CI [0.20, 1.07], I2 = 29.4% as shown in Figure 5. The regression test and the rank correlation indicated no publication bias, p = 0.99, p = 0.98, respectively.

### Amh Level According to Vitamin D Level

Analysis of findings extracted from 4 studies weighing the impact of vitamin D status (25(OH) Vitamin D < 20 ng/ml vs. 25(OH) Vitamin D > 20 ng/ml) on AMH level. Power of sufficient Vitamin D level was found to be non-significant (p < 0.25) on the level of AMH, MD = –2.39 95%CI [–6.47, 1.68], I2 = 99.7% as shown in Supplementary Figure S1a. The regression test and (p = 0.08) indicated no publication bias, while the rank correlation (p < 0.001) showed funnel plot asymmetry.

### Effects of Vitamin D Supplementation on Serum Amh Levels

Analysis of findings extracted from 8 studies assessing the influence of vitamin D supplement on serum vitamin D levels compared to baseline levels before initiation of therapy. Impact of Vitamin D supplement was found to be non-significant (p < 0.79) on serum levels of AMH post-treatment compared with baseline values, MD = –0.08, 95%CI [–0.68, 0.52], I2 = 93.1% as shown in Supplementary Figure S1b. Both regression test and rank correlation indicated potential funnel plot asymmetry (p = 0.0016 and p = 0.0247, respectively).

## DISCUSSION

Following an examination of 2111 relevant studies, the meta-analysis comprised 22 publications spanning the years 2009 to 2023^(20,28,29,30,31,32,32,33,34,35,36,37,38,39,40,41,42,43,44,45,46,47,48)^. These articles were selected for inclusion because they satisfied the inclusion criteria. Figures 2-5 contain a compilation of the findings that were obtained from these investigations.

A significant improvement was observed in several parameters, including the level of vitamin D, the number of dominant follicles, the ovulation rate, and the pregnancy rate (mean difference = –2.32 95% confidence interval [–3.07, –1.56], mean difference = 0.81 95% confidence interval [0.27, 1.35], mean difference = 0.81 95% confidence interval [0.17, 1.45], and mean difference = 0.64 95% confidence interval [0.20, 1.07], respectively). On the other hand, there was no discernible effect on the levels of AMH.

Supplementing with vitamin D enhanced the rate of ovulation in females with PCOS. Berry et al. assert that 70% of infertile females with PCOS exhibit a deficiency in vitamin D compared to healthy females^(49)^. Moreover, studies reveal that there is a direct correlation between vitamin D insufficiency and the quantity of fully developed eggs^(50)^ as well as the frequency of ovulation^(51,52)^. However, various concepts continue to exist. One study indicates that the levels of serum vitamin D in females with PCOS are comparable to those without the condition^(53)^. The difference may stem from various ethnicities and the particular subtype of PCOS. Besides the vitamin D receptor and the mechanism associated with sex hormones, the main way in which vitamin D affects ovulation is by downregulating the AMH receptor II. This, in turn, promotes the activation and maturation of oocytes^(54)^.

Taking vitamin D supplements clearly increased the number of pregnancies among females. Other measures related to assisted reproductive technology, including the rates of fertilization, cleavage, and high-quality embryos, were unaffected. No extensive study on the length of pregnancy in PCOS females who have used vitamin D supplements has been found. Present study mainly examines the correlation between vitamin D levels and in vitro fertilization success rates; however, the results are not conclusive. To be more specific, the levels of vitamin D in the blood and follicular fluid may increase, decrease, or remain unchanged, and the rate of conception. Vitamin D levels vary from patient to patient, which could explain the discrepancy in results. There has not been a comprehensive investigation of the exact process by which vitamin D increases the likelihood of conception in PCOS females. We assumed that vitamin D supplementation may improve fertility in PCOS females based on the results of this study. Improving ovulation, thickening the endometrial lining, and decreasing inflammation in the granulosa cells could all contribute to this goal^(55)^.

A previous study by Yang et al.^(56)^ indicated that in PCOS patients compared to the control group, the vitamin D intervention group had noticeably reduced levels of luteinizing hormone (LH) and FSH. The results suggest that taking vitamin D supplements may lower LH levels when using short-acting contraceptives or ovulation-stimulating medications. There is a negative association between blood vitamin D levels and LH and FSH levels, according to recent findings. But there was no statistically significant difference^(57)^. This study focuses on females with PCOS, in contrast to previous studies that found no correlation between LH, FSH, and vitamin D levels. This discrepancy could be because previous studies mostly included healthy reproductive-age females.

Regarding the interventional studies analyzed in this meta-analysis, several studies found that non-PCOS females with vitamin D deficiency experienced a rise in serum AMH levels after taking both short-term and long-term vitamin D supplements. Unlike Cappy et al.^(33)^ study which did not observe any alterations in serum AMH levels after administering vitamin D supplements to women with either PCOS or without PCOS. One potential explanation for this difference could be attributed to the effectiveness of vitamin D supplements. The average vitamin D levels after treatment in the study conducted by Cappy et al. were slightly above the insufficient range (31.1 ± 8.5 and 32.0 ± 9.2 ng/mL in the control and PCOS women, respectively), which is significantly lower compared to the levels observed in other studies. Two more interventional studies conducted by Irani et al.^(19)^ and Dastorani et al.^(31)^ examined females with PCOS and found that vitamin D supplements specifically reduced AMH levels in this population^(31,35)^.

## LIMITATIONS

There are a few restrictions that apply to this review. The first is the quality of the trials, which are fraught with a high risk of bias, inconsistency, and imprecision, serves to restrict the certainty of the findings. It is important to handle certain restrictions with caution. The second issue is that the included studies might have been of better quality. Additionally, certain indicators ’ data, such as the cumulative pregnancy rate, were not made public, which limits the scope of the investigation.

## CONCLUSION

Vitamin D therapy appears to have a positive impact on reproductive outcomes, according to the findings of recent research. These outcomes include the number of dominant follicles, the rate of ovulation, and the rate of pregnancy. However, in order to provide support for these findings, large-scale randomized trials that involve several centers are required.

## Data Availability

The entire dataset supporting the results of this study is available upon request to corresponding author.

## SUPPLEMENTARY MATERIAL

The following online material is available for this article:

Figure S1 – Forrest plot assessing the AMH Level According to Vitamin D Level (a) and Effects of Vitamin D Supplementation on Serum AMH Levels (b).
