# Supplementary material for: A meta-analysis of the literature evaluating the impact of vitamin D on female fertility and ovarian reserve function
Source: Rev Esc Enferm USP. 2026 Feb 2;60:e20250382. doi: 10.1590/1980-220X-REEUSP-2025-0382en (PMC12893472; doi:10.1590/1980-220X-REEUSP-2025-0382en)
Supplement: Supplementary file 1 [file 1980-220X-reeusp-60-e20250382-suppl1.pdf]

Rev Esc Enferm USP
https://doi.org/10.1590/1980-220X-REEUSP-2025-0382en

Supplementary Material to "A meta-analysis of the literature evaluating the impact of vitamin D on female fertility and ovarian reserve function"

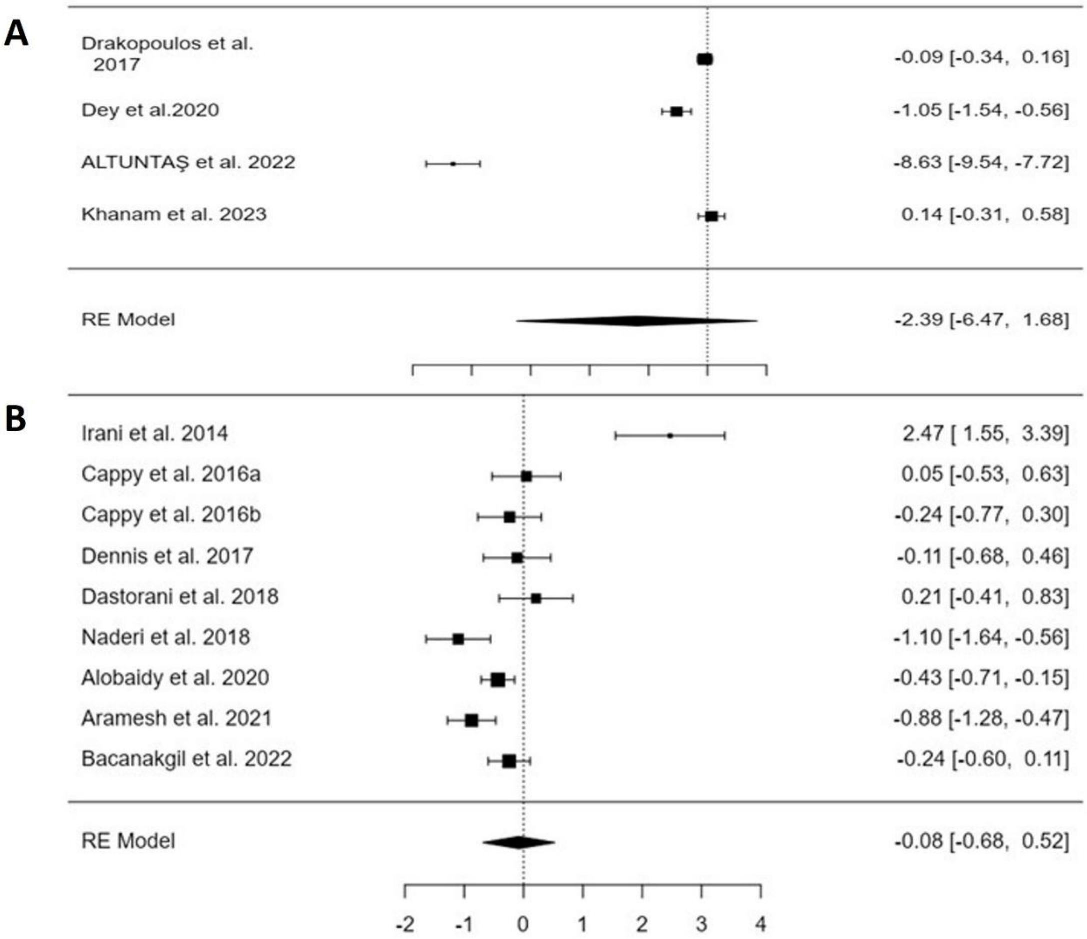

Figure S1 – Forrest plot assessing the AMH Level According to Vitamin D Level (a) and Effects of Vitamin D Supplementation on Serum AMH Levels (b).
