# Supplementary material for: A meta-analysis of the literature evaluating the impact of vitamin D on female fertility and ovarian reserve function
Source: Rev Esc Enferm USP. 2026 Feb 2;60:e20250382. doi: 10.1590/1980-220X-REEUSP-2025-0382en (PMC12893472; doi:10.1590/1980-220X-REEUSP-2025-0382en)
Supplement: Translation

# Translation: Metaanálisis de la literatura que evalúa el impacto de la vitamina D en la fertilidad femenina y la función de reserva ovárica

**DOI:** 10.1590/1980-220X-REEUSP-2025-0382es

**Authors:** Shanfei Zhao, Wenling Zheng, Guanyun Long, Guanglin Liang

**Affiliations:** 1 Maoming People ’s Hospital, Center of Reproductive Medicine, Guangdong Province, China.

**Keywords:** Vitamina D, Infertilidad, Síndrome del Ovario Poliquístico, Ovulación; Embarazo, Hormona Antimülleriana

## Abstract

**Objetivo:**

El presente estudio de metaanálisis tiene como objetivo estimar el papel de los niveles de vitamina D y de la suplementación con vitamina D en la reserva reproductiva, expresada por la tasa de ovulación, la tasa de embarazo y la hormona anti-Mülleriana (AMH).

**Método:**

Se llevó a cabo una revisión sistemática de la literatura y se reclutaron 2.111 mujeres en el estudio actual. Los criterios de inclusión comprendieron estudios comparativos que evaluaron el estado de vitamina D y la suplementación con vitamina D en parámetros de fertilidad. Se incluyeron estudios publicados hasta diciembre de 2023. Para el análisis de los datos se utilizó un modelo en modalidad continua con efectos aleatorios.

**Resultados:**

Se incluyeron veintidós estudios que investigaron el impacto de la vitamina D en los parámetros de fertilidad femenina. La suplementación con vitamina D mostró una mejora significativa en varios parámetros, incluyendo el nivel de vitamina D, el número de folículos dominantes, la tasa de ovulación y la tasa de embarazo (MD = –2.3295; 95% CI [–3.07, –1.56]; MD = 0.81; 95% CI [0.27, 1.35]; MD = 0.81; 95% CI [0.17, 1.45]; y MD = 0.64; 95% CI [0.20, 1.07], respectivamente). Por el contrario, no se observó un impacto significativo sobre los niveles de AMH.

**Conclusión:**

Los hallazgos del presente estudio indican un efecto beneficioso de la terapia con vitamina D en los resultados de fertilidad en lo que respecta al número de folículos dominantes, la tasa de ovulación y la tasa de embarazo.

## INTRODUCCIÓN

La infertilidad afecta al 75 % de las mujeres con síndrome de ovario poliquístico (SOP), lo que lo convierte en el trastorno endocrino más común que afecta a las mujeres en edad reproductiva^(1)^. Monitorear la ovulación, estimular la ovulación, la perforación ovárica y el uso de técnicas de reproducción asistida son las principales formas actuales de tratar la infertilidad en el SOP^(2)^. Varios nutrientes, como los minerales, las vitaminas y los ácidos grasos insaturados, también han suscitado mucho interés cuando se utilizan como coadyuvantes^(3)^. La deficiencia de vitamina D es un problema grave que afecta al 67-85 % de las mujeres con SOP^(4)^. Las pacientes con SOP pueden desarrollar resistencia a la insulina y síndrome metabólico. La insuficiencia de vitamina D puede contribuir a ello^(5)^. Las mujeres embarazadas con SOP están experimentando una ligera tendencia al alza en las investigaciones sobre la eficacia de los suplementos de vitamina D. Los efectos protectores de la vitamina D contra el estrés oxidativo y su papel en la regulación de los niveles de iones de calcio, que son fundamentales para preservar el potencial de reposo de las células, son las razones que explican esta tendencia. Los procesos de fertilización, segmentación, implantación y desarrollo placentario pueden beneficiarse de estos efectos^(6)^.

Nuevos estudios en animales y seres humanos sugieren que la vitamina D interviene en la fisiología de la reproducción femenina^(7)^, aunque no se conoce bien el mecanismo específico. Las ratas con una dieta insuficiente en vitamina D experimentan una disminución del 75 % en la fertilidad, acompañada de una disminución del 30 % en el número de crías y un retraso en el crecimiento de los recién nacidos^(8)^. Los ratones knockout para el receptor de vitamina D mostraron una reducción de la formación ósea y un retraso en el crecimiento, así como un útero subdesarrollado y una disminución del desarrollo folicular^(9,10)^. Los ratones presentaban hipogonadismo hipergonadotrópico, que se complementaba con una reducción de la expresión del gen CYP19 y de la actividad de la aromatasa. Esto sugiere que la vitamina D podría tener un papel protagonista en la producción de estrógenos y en la expresión del gen de la aromatasa^(9,10)^. Los ratones que reciben 25-hidroxivitamina D 1a-hidroxilasa presentan anomalías comparables, como un útero subdesarrollado, una disminución del crecimiento de los folículos ováricos y una formación defectuosa del cuerpo lúteo. Cabe destacar que la administración de una dieta rica en calcio y/o fósforo restaura eficazmente la fertilidad de estos animales. Esto indica que la infertilidad en estos modelos es el resultado de los bajos niveles de calcio y/o fósforo causados por una deficiencia de vitamina D^(11,12)^. Cuando se aplicó vitamina D a las células ováricas en un entorno de laboratorio, se produjo un aumento de la producción de las hormonas sexuales progesterona, estrógeno y estrona^(13)^. Además, la placenta humana experimentó un aumento en la síntesis de estrógeno y progesterona cuando se expuso a 1,25-dihidroxivitamina D3^(14)^. Además, un gran número de estudios indican que existe una correlación entre la insuficiencia de vitamina D y otros síntomas del SOP, como la anovulación, el hiperandrogenismo y la resistencia a la insulina^(15)^. Cabe señalar que las mujeres con SOP son más propensas a tener una deficiencia de vitamina D^(16,17)^. Los estudios han demostrado que la suplementación con vitamina D puede mejorar la ciclicidad menstrual, reducir el hiperan-drogenismo y mejorar los factores metabólicos asociados a este síndrome^(18,19)^. Esto sugiere que la vitamina D beneficia directamente la fertilidad femenina.

No obstante, los hallazgos existentes sobre el impacto de los suplementos de vitamina D en las tasas de embarazo carecen de consistencia. Varios estudios han demostrado resultados diferentes con respecto al impacto de los suplementos de vitamina D en las tasas de embarazo en mujeres con SOP. Algunos estudios han indicado un efecto positivo, mientras que otros no han encontrado ningún beneficio sustancial^(20)^. El objetivo de este estudio es recopilar y analizar de forma sistemática las pruebas existentes sobre el uso de la vitamina D como tratamiento complementario para la infertilidad en las mujeres. El objetivo del análisis del presente estudio es determinar si la vitamina D puede mejorar los resultados del embarazo, como la tasa de embarazo, la tasa de ovulación, el nivel de hormona antimülleriana (Anti-Müllerian Hormone - AMH) y el número de folículos dominantes en las mujeres, y sentar las bases para la medicación clínica.

## MÉTODO

### Diseño del Estudio

En el presente metaanálisis se utilizó la declaración epidemiológica, que abarcaba estudios que seguían una técnica de estudio preestablecida^(21)^. Se utilizaron varias bases de datos científicas para obtener y analizar los datos de los estudios reclutados. Estas bases de datos científicas incluían OVID, Cochrane Library, PubMed, Embase y Google Scholar. Se siguieron con precisión los criterios de inclusión^(22)^. La secuencia completa del estudio se muestra en la Figura 1.

**Figura 1:** Diagrama que representa el procedimiento del estudio – Guangdong, China, 2025.

### Elegibilidad e Inclusión

Los estudios incluidos deben estar orientados a evaluar el papel de los niveles de vitamina D y los suplementos en la reserva de fertilidad expresada como tasa de ovulación, tasa de embarazo y hormona antimülleriana. Solo se incluyeron en el análisis de sensibilidad los artículos que evaluaban específicamente las intervenciones que afectaban a la tasa de ovulación, la tasa de embarazo y los niveles de vitamina D, así como la forma en que los niveles de vitamina D y los suplementos afectaban a la reserva de fertilidad, medida por el recuento de folículos dominantes, la tasa de ovulación, la tasa de embarazo y el nivel de hormona antimulleriana^(23)^.

### Criterios de Inclusión:

1. El diseño de estudio aceptable para participar en el presente estudio debe ser un estudio comparativo publicado antes de diciembre de 2024.
2. Los estudios de mujeres que recibieron terapia de suplementos de vitamina D en comparación con mujeres de control tienen niveles séricos previos y posteriores de AMH y vitamina D.

### Los Criterios de Exclusión Fueron:

1. Artículos que no pudieron transmitir los resultados de la comparación entre diferentes intervenciones de manera adecuada, como el rango intercuartílico o la mediana. Los estudios siempre deben proporcionar sus datos como media (DE) o evento/total.
2. En caso de que el estudio fuera en forma de cartas, libros, artículos de revisión o capítulos de libros.

### Identificación

En primer lugar, hasta diciembre de 2024, utilizamos una combinación de varias palabras clave y palabras comparables para la suplementación con vitamina D, el nivel de vitamina D, la AMH, la hormona folículoestimulante (FSH), la fertilidad, la tasa de ovulación, la tasa de embarazo, el SOP y los folículos^(24)^. Se definió un protocolo de estrategias de búsqueda de acuerdo con el principio PICOS de la siguiente manera: P (población), mujeres con problemas relacionados con la fertilidad; I (intervención/exposición), suplementos de vitamina D; C (comparación), grupo de intervención con grupo de control o comparación antes y después de la intervención; O (resultado), nivel de vitamina D, nivel de AMH, tasa de ovulación, tasa de embarazo, número de folículos dominantes; S (diseño del estudio): estudios comparativos intervencionistas.

Utilizando estas palabras clave y otras asociadas, los autores realizaron búsquedas en Google Scholar, PubMed, Cochrane Library, Embase y OVID hasta diciembre de 2024. Tras revisar los títulos y resúmenes incluidos en el programa de gestión de referencias, se excluyó cualquier artículo que no abordara y evaluara el impacto de la vitamina D en los parámetros reproductivos^(25)^.

### Selección

Se utilizaron varios criterios para filtrar los datos. Entre ellos se encontraban los siguientes: el apellido del primer autor, el año de publicación, el país del estudio, el tipo de estudio, la duración del estudio, la información demográfica, las características clínicas y del tratamiento, el número total de sujetos, los métodos utilizados, la fuente de información y los resultados. Dos autores independientes examinaron de forma ciega la calidad metodológica de los artículos seleccionados y verificaron cada estudio en busca de posibles sesgos^(26)^.

Cada investigación incluida se evaluó en busca de posibles sesgos utilizando un gestor de autores, y los resultados se clasificaron de menor a mayor. Cada estudio fue sometido a un examen metodológico por parte de dos autores independientes^(27)^.

### Análisis Estadístico

En este metaanálisis, utilizamos un efecto aleatorio con modelo continuo para calcular la diferencia media (DM) con un intervalo de confianza (IC) del 95 %. 24 Este estudio utilizó pruebas bilaterales para obtener todos los p-valores. Los gráficos y los análisis estadísticos se crearon utilizando Jamovi. Utilizando el estimador de máxima verosimilitud restringida, se calculó el nivel de heterogeneidad (tau^2^). Se calculó el índice I^2^, que es un valor numérico con un rango de 0 a 100 y se transmite en forma de diagramas de bosque. El nivel de heterogeneidad se mostró mediante porcentajes que oscilaban entre el 0 % y el 100 %, y también se expresó mediante porcentajes que indicaban niveles de heterogeneidad nulos (<25 %), bajos (<50 %), moderados (<75 %) y altos. Se utilizaron las pruebas de Begg y Egger para realizar un análisis cuantitativo del sesgo de publicación, y se consideró que existía sesgo de publicación si p > 0,05.

## RESULTADOS

Tras revisar 2111 estudios relevantes, se incluyeron en el metaanálisis 22 artículos del periodo comprendido entre 2009 y 2023, ya que cumplían los criterios de inclusión^(20,28,29,30,31,32,32,33,34,35,36,37,38,39,40,41,42,43,44,45,46,47,48)^. Los resultados de estas investigaciones se recogen en las Figuras 2 a 5.

### Influencia de los Suplementos de Vitamina D en los Niveles Séricos de Vitamina D

Análisis de los resultados extraídos de nueve estudios que evaluaron la influencia de los suplementos de vitamina D en los niveles de vitamina D en comparación con los niveles basales antes del inicio del tratamiento. Se ha revelado que el impacto de la vitamina D es significativo (p < 0,001) en los niveles séricos de vitamina D después del tratamiento en comparación con los valores basales, DM = –2,32, IC del 95 % [-3,07, –1,56], I2 = 95,2 %, como se muestra en la Figura 2. La prueba de regresión indicó una asimetría del gráfico de embudo (p = 0,08), mientras que la correlación de rangos no indicó sesgo de publicación (p = 0,73).

**Figura 2:** Forest plot que indica la influencia del tratamiento con vitamina D en el nivel de vitamina D sérica – Guangdong, China, 2025.

### Número de Folículos Dominantes

Análisis de los resultados extraídos de siete artículos que evalúan la influencia de los suplementos de vitamina D en el número de folículos dominantes en comparación con el grupo de control (grupo de mujeres que no recibieron suplementos de vitamina D). Se ha revelado que el impacto de la vitamina D es significativo (p < 0,01) en el número de folículos dominantes, expresado como un número elevado de folículos dominantes en las mujeres que reciben suplementos de vitamina D en comparación con el control, DM = 0,81, IC del 95 % [0,27, 1,35], I2 = 1,18 %, como se muestra en la Figura 3. La prueba de regresión y la correlación de rangos no indicaron sesgo de publicación, p = 0,1, p = 0,24, respectivamente.

**Figura 3:** Forest plot que indica la influencia del tratamiento con vitamina D en el número de folículos dominantes – Guangdong, China, 2025.

### Tasa de Ovulación

Análisis de los resultados extraídos de cinco estudios que evalúan la influencia de los suplementos de vitamina D en la tasa de ovulación en comparación con el grupo de control. Se ha revelado que el impacto de la vitamina D es significativo (p = 0,01) en la tasa de ovulación, expresado como una alta tasa de ovulación en las mujeres que reciben suplementos de vitamina D en comparación con el control, DM = 0,81, IC del 95 % [0,17, 1,45], I2 = 5,1 %, como se muestra en la Figura 4. La prueba de regresión y la correlación de rangos no indicaron ningún sesgo de publicación, p = 0,52, p = 0,82, respectivamente.

**Figura 4:** Forest plot que indica la influencia del tratamiento con vitamina D en la tasa de ovulación – Guangdong, China, 2025.

### Tasa de Embarazo

Análisis de los resultados extraídos de nueve estudios que evalúan el impacto de los suplementos de vitamina D en la tasa de embarazo en comparación con la del grupo de control. El impacto de la vitamina D fue significativo (p < 0,01) en la tasa de embarazo, expresada como una alta tasa de embarazo en las mujeres que recibieron suplementos de vitamina D en comparación con el grupo de control, DM = 0,46, IC del 95 % [0,20, 1,07], I2 = 29,4 %, como se muestra en la Figura 5. La prueba de regresión y la correlación de rangos no indicaron sesgo de publicación, p = 0,99, p = 0,98, respectivamente.

**Figura 5:** Forest plot que indica la influencia del tratamiento con vitamina D en la tasa de embarazo – Guangdong, China, 2025.

### Nivel de Amh Según el Nivel de Vitamina D

Análisis de los resultados extraídos de cuatro estudios que evalúan el impacto del estado de la vitamina D (25(OH) vitamina D < 20 ng/ml frente a 25(OH) vitamina D > 20 ng/ml) en el nivel de AMH. Se observó que el poder de un nivel suficiente de vitamina D no era significativo (p < 0,25) en el nivel de AMH, DM = –2,39, IC del 95 % [–6,47, 1,68], I2 = 99,7 %, como se muestra en la Figura Suplementaria S1a. La prueba de regresión (p = 0,08) no indicó ningún sesgo de publicación, mientras que la correlación de rangos (p < 0,001) mostró una asimetría en el gráfico de embudo.

### Efectos de Los Suplementos de Vitamina D en los Niveles Séricos de Amh

Análisis de los resultados extraídos de ocho estudios que evaluaron la influencia de los suplementos de vitamina D en los niveles séricos de vitamina D en comparación con los niveles basales antes del inicio del tratamiento.

Se observó que el impacto de los suplementos de vitamina D no era significativo (p-valor < 0,79) en los niveles séricos de AMH después del tratamiento en comparación con los valores basales, DM = –0,08, IC del 95 % [–0,68, 0,52], I2 = 93,1 %, como se muestra en la Figura Suplementaria S1b. Tanto la prueba de regresión como la correlación de rangos indicaron una posible asimetría en el gráfico de embudo (p = 0,0016 y p = 0,0247, respectivamente).

## DISCUSIÓN

Tras examinar 2111 estudios relevantes, el metaanálisis incluyó 22 publicaciones que abarcaban los años 2009 a 2023^(20,28,29,30,31,32,32,33,34,35,36,37,38,39,40,41,42,43,44,45,46,47,48)^. Estos artículos se seleccionaron para su inclusión porque cumplían los criterios de inclusión. Las Figuras 2-5 contienen una recopilación de los resultados obtenidos en estas investigaciones.

Se observó una mejora significativa en varios parámetros, entre ellos el nivel de vitamina D, el número de folículos dominantes, la tasa de ovulación y la tasa de embarazo (diferencia media = –2,32, intervalo de confianza del 95 % [–3,07, –1,56], diferencia media = 0,81, intervalo de confianza del 95 % [0,27, 1,35], diferencia media = 0,81, intervalo de confianza del 95 % [0,17, 1,45] y diferencia media = 0,64, intervalo de confianza del 95 % [0,20, 1,07], respectivamente). Por otro lado, no se observó ningún efecto apreciable en los niveles de AMH.

La suplementación con vitamina D mejoró la tasa de ovulación en mujeres con SOP. Berry et al. afirman que el 70 % de las mujeres infértiles con SOP presentan una deficiencia de vitamina D en comparación con las mujeres sanas^(49)^. Además, los estudios revelan que existe una correlación directa entre la insuficiencia de vitamina D y la cantidad de óvulos completamente desarrollados^(50)^, así como la frecuencia de la ovulación^(51,52)^. Sin embargo, siguen existiendo diversos conceptos. Un estudio indica que los niveles de vitamina D sérica en mujeres con SOP son comparables a los de las mujeres que no padecen esta afección^(53)^. La diferencia puede deberse a las diversas etnias y al subtipo particular de SOP. Además del receptor de vitamina D y el mecanismo asociado a las hormonas sexuales, la principal forma en que la vitamina D afecta a la ovulación es mediante la regulación a la baja del receptor II de AMH. Esto, a su vez, promueve la activación y maduración de los ovocitos^(54)^.

La toma de suplementos de vitamina D aumentó claramente el número de embarazos entre las mujeres. Otras medidas relacionadas con la tecnología de reproducción asistida, como las tasas de fertilización, división y embriones de alta calidad, no se vieron afectadas. No se ha encontrado ningún estudio exhaustivo sobre la duración del embarazo en mujeres con SOP que han tomado suplementos de vitamina D. El presente estudio examina principalmente la correlación entre los niveles de vitamina D y las tasas de éxito de la fertilización in vitro; sin embargo, los resultados no son concluyentes. Para ser más específicos, los niveles de vitamina D en la sangre y el líquido folicular pueden aumentar, disminuir o permanecer sin cambios, al igual que la tasa de concepción. Los niveles de vitamina D varían de una paciente a otra, lo que podría explicar la discrepancia en los resultados. No se ha realizado una investigación exhaustiva sobre el proceso exacto por el cual la vitamina D aumenta la probabilidad de concepción en mujeres con SOP. Supusimos que los suplementos de vitamina D podrían mejorar la fertilidad en mujeres con SOP basándonos en los resultados de este estudio. La mejora de la ovulación, el engrosamiento del revestimiento endometrial y la disminución de la inflamación en las células de la granulosa podrían contribuir a este objetivo^(55)^.

Un estudio anterior de Yang et al.^(56)^ indicó que, en las pacientes con SOP, en comparación con el grupo de control, el grupo de intervención con vitamina D había reducido notablemente los niveles de hormona luteinizante (LH) y FSH. Los resultados sugieren que tomar suplementos de vitamina D puede reducir los niveles de LH cuando se utilizan anticonceptivos de acción corta o medicamentos para estimular la ovulación. Según hallazgos recientes, existe una asociación negativa entre los niveles de vitamina D en sangre y los niveles de LH y FSH. Sin embargo, no se observaron diferencias estadísticamente significativas^(57)^. Este estudio se centra en mujeres con SOP, a diferencia de estudios previos que no encontraron ninguna correlación entre los niveles de LH, FSH y vitamina D. Esta discrepancia podría deberse a que los estudios previos incluían principalmente a mujeres sanas en edad reproductiva.

En cuanto a los estudios intervencionistas analizados en este metaanálisis, varios estudios encontraron que las mujeres sin SOP con deficiencia de vitamina D experimentaron un aumento en los niveles séricos de AMH después de tomar suplementos de vitamina D a corto y largo plazo. A diferencia del estudio de Cappy et al.^(33)^, que no observó ninguna alteración en los niveles séricos de AMH después de administrar suplementos de vitamina D a mujeres con o sin SOP. Una posible explicación de esta diferencia podría atribuirse a la eficacia de los suplementos de vitamina D. Los niveles medios de vitamina D después del tratamiento en el estudio realizado por Cappy et al.^(33)^ estaban ligeramente por encima del rango insuficiente (31,1 ± 8,5 y 32,0 ± 9,2 ng/mL en las mujeres del grupo de control y con SOP, respectivamente), lo que es significativamente inferior en comparación con los niveles observados en otros estudios. Otros dos estudios intervencionales realizados por Irani et al.^(19)^ y Dastorani et al.^(31)^ examinaron a mujeres con SOP y descubrieron que los suplementos de vitamina D reducían específicamente los niveles de AMH en esta población^(31,35)^.

## LIMITACIONES

Hay algunas restricciones que se aplican a esta revisión. La primera es la calidad de los ensayos, que están plagados de un alto riesgo de sesgo, inconsistencia e imprecisión, lo que limita la certeza de los resultados. Es importante manejar ciertas restricciones con precaución. La segunda cuestión es que los estudios incluidos podrían haber sido de mejor calidad. Además, los datos de ciertos indicadores, como la tasa acumulada de embarazos, no se hicieron públicos, lo que limita el alcance de la investigación.

## CONCLUSIÓN

Según los resultados de investigaciones recientes, el tratamiento con vitamina D parece tener un impacto positivo en los resultados reproductivos. Estos resultados incluyen el número de folículos dominantes, la tasa de ovulación y la tasa de embarazo. Sin embargo, para respaldar estos resultados, se requieren ensayos aleatorios a gran escala en los que participen varios centros.

## Data Availability

El conjunto de datos que respaldan las conclusiones de este estudio está disponible previa solicitud al autor correspondiente.

## MATERIAL SUPLEMENTARIO

El siguiente material suplementario está disponible en línea:

Figura S1 – Forrest plot assessing the AMH Level According to Vitamin D Level (a) and Effects of Vitamin D Supplementation on Serum AMH Levels (b).
